# Serologic Evidence of West Nile Virus Infection among Humans, Morocco

**DOI:** 10.3201/eid1805.110826

**Published:** 2012-05

**Authors:** Hicham El Rhaffouli, Mehdi El Harrak, Chafiqa Lotfi, Fatima El Boukhrissi, Tahar Bajjou, Abdelilah Laraqui, Farida Hilali, Mouna Kenfaoui, Idriss Lahlou-Amine

**Affiliations:** University Mohammed V-Souissi, Rabat, Morocco (H. El Rhaffouli, F. El Boukhrissi, T. Bajou, A. Laraqui, F. Hilali, I. Lahlou-Amine);; Laboratoire Bio-Pharma, Rabat (M. El Harrak, C. Lotfi);; Military Hospital Moulay Ismail, Meknes, Morocco (F. El Boukhrissi);; Laboratoire Maamora, Kenitra, Morocco (M. Kenfaoui)

**Keywords:** West Nile virus, neutralizing antibody, virus-neutralization test, human, Morocco, viruses, transmission

**To the Editor**: West Nile virus (WNV) infections were reported in horses in Morocco in 1996, 2003 (*1*), and 2010 (*2*). The isolates from 1996 and 2003 belong to WNV lineage 1, clade 1a (*1*). In 1996, WNV infection was reported in a human in Morocco (*3*), and in 2008, a serosurvey of wild birds confirmed the circulation of WNV in native birds (*4*). To our knowledge, there are no seroprevalence data for WNV antibodies in humans in Morocco. Thus, we evaluated the prevalence of WNV neutralizing bodies in serum samples collected during March–April 2011 from 499 healthy persons living in the vicinities of Meknes, Rabat, or Kenitra. All persons consented to study participation.

The participants were divided into 3 cohorts, A, B, and C. Cohort A consisted of 150 persons from the Meknes area, where no WNV infections among horses have been reported. The mean age of persons in cohort A was 52 years (SD ± 15 years), and 31% were male. Cohort B consisted of 200 persons living in the region of Rabat (median age 49 years [SD ± 12 years]; 38% male), where the WNV outbreaks among horses were described in 1996 (*3*) and 2010 (*2*). Cohort C consisted of 149 participants living in the region of Kenitra (median age 48 years [SD ± 17 years]; 43% male), which was affected by the WNV outbreaks among horses in 1996, 2003, and 2010.

Serum was stored at −20°C until tested. Just before testing, serum samples were heated at 56°C for 30 minutes. The samples were screened for neutralizing antibody against the equine WNV strain, Morocco 96–111 (*3*), by using a micro virus-neutralization test in 96-well plates and an adaptation of a described method (*5*). Dilutions of test serum (50 µL) were incubated with one hundred 50% tissue culture infectious doses of the virus in the same volume (50 μL) for 1 hour at 37°C in Dulbecco minimum Eagle medium. We then added 150 μL (10^5^ cells/mL) of a Vero cell suspension with 5% fetal calf serum. This mixture was incubated for 5–6 days at 37°C until cytopathic effects were observed in a negative control well containing a 50% tissue culture infectious dose of virus. Serum samples were screened in duplicate at dilutions of 1:6, 1:18, and 1:54. Samples that neutralized the virus, characterized by absence of cytopathic effects, at 1 of the dilutions tested were retested in 4 replicates to confirm the result. We then titrated the samples by testing 6 serial dilutions ranging from 1:6 to 1:1,458. Titers were calculated by using the Spearman-Kärber method (*6*). Titers >18 were considered positive.

Of the 499 participants, 59 (11.8%) had WNV neutralizing bodies (7 of 150 in cohort A, 24 of 200 in cohort B, and 28 of 149 in cohort C). Titers determined by the micro virus-neutralization test ranged from 18 to 2,630 (Table).The prevalence of WNV neutralizing bodies was significantly higher in cohort B and C participants than in cohort A participants (p<0.01). A significant correlation was not observed between the presence of WNV neutralizing bodies and the age or sex of participants.

**Table:** West Nile virus neutralizing antibody titers in human serum samples obtained during March–April 2011, Morocco

Cohort, location*	No. samples positive/no. tested (%)	Median ± SD	Titer (%)		
			Weak, 18–80	Intermediate, 81–320	High, >320
A, Meknes	7/150 (4.7)	54 ± 42.5	5 (71.4)	1 (14.3)	1 (14.3)
B, Rabat	24/200 (12)	54 ± 31	22 (91.7)	1 (4.2)	1 (4.2)
C, Kenitra	28/149 (18.8)	95 ± 72	19 (67.9)	3 (10.7)	6 (21.4)
Total	59/499 (11.8)	72 ± 41	46 (78)	5 (8.5)	9 (13.5)

*Study participants were divided into 3 cohorts according to the vicinity from which they were recruited.

The low prevalence of WNV neutralizing antibodies (4.7%; median ± SD titer 54 ± 42.5) in persons from Meknes (cohort A) suggests a low level of WNV circulation in the area. This finding is likely related to the ecosystem of this region (arid and semi-arid, with an average altitude of 500 m), which is unfavorable for survival of the vectors. Infections are more likely the result of travel to areas where WNV is endemic.

In persons from Rabat (cohort B), the medium prevalence reported (12%; median ± SD titer 54 ± 31) confirms human infection with epidemic WNV strains found in horses during WNV epidemics of 1996 and 2010. However, this prevalence is lower than that for persons in Kenitra (cohort C) (18.8%; median titer 95 ± 72). High titers were obtained in cohort C, which is located in an extremely humid area that includes several wetlands and rice fields flooded through regulated channels from the Sebou River. In addition, this region includes 2 natural bird reserves along a principal migratory Europe–sub-Saharan route. These conditions are favorable for WNV circulation and are likely related to the high prevalence of WNV infection registered in this region.

We found evidence for local circulation of WNV in Morocco. The Moroccan WNV strains most often cause mild and self-limiting illnesses. These illnesses are difficult to distinguish from many other febrile illnesses, making it less likely that viral testing would be performed for WNV. Our results show that WNV is an emerging disease in Morocco, and a national response plan should be implemented by public health authorities.

## References

[1] Schuffenecker I, Peyrefitte CN, El Harrak M, Murri S, Leblond A, Zeller H. West Nile virus in Morocco, 2003. Emerg Infect Dis. 2005;11:306–9. 10.3201/eid1102.040817 15752452 PMC3320441

[2] World Animal Health Information Database. Event summary: West Nile fever, Morocco. 2010 Sep [cited 2011 Apr 10]. http://web.oie.int/wahis/public.php?page=event_summary&reportid=9615

[3] El Harrak M, Le Guenno B, Le Gounon P. Isolation of West Nile virus in Morocco [in French]. Virologie. 1997;1:248–9 [cited 2011 May 20]. http://www.jle.com/fr/revues/bio_rech/vir/e-docs/00/03/F8/1C/article.md?type=text.html

[4] Figuerola J, Baouab RE, Soriguer R, Fassi-Fihri O, Llorente F, Jimenez-Clavero MA. West Nile virus antibodies in wild birds, Morocco, 2008. Emerg Infect Dis. 2009;15:1651–3. 19861065 10.3201/eid1510.090340 PMC2866403

[5] Weintgartl HM, Drebot MA, Hubalek Z, Halouzka J, Andonova M, Dibernardo A, Comparison of assays for the detection of West Nile virus antibodies in chicken serum. Can J Vet Res. 2003;67:128–32 [cited 2011 May 15]. http://www.ncbi.nlm.nih.gov/pmc/articles/PMC227040/?tool=pubmed PMC227040 12760478

[6] Kärber G. Beitrag zur kollektiven Behandlung pharmakologischer Reihenversuche. Arch Pharmacol. 1931;162:480–3.

